# *Ex Vivo* Evaluation of Secretion-Clearing Device in Reducing Airway Resistance within Endotracheal Tubes

**DOI:** 10.1155/2018/3258396

**Published:** 2018-12-10

**Authors:** Christopher Waters, R. Constance Wiener, Hamed M. Motlagh

**Affiliations:** ^1^Department of Dental Research, West Virginia University, Health Sciences Addition Room 106a, PO Box 9448, Morgantown, WV 26506, USA; ^2^Dental Practice and Rural Health, West Virginia University, Health Sciences Addition Room 104a, PO Box 9448, Morgantown, WV 26506, USA; ^3^School of Dentistry, West Virginia University, Morgantown, WV, USA

## Abstract

**Background:**

Secretions accumulate in endotracheal tubes' (ETT) lumens upon their placement in patients. The secretions impact airway resistance and pressure. Secretions potentiate prolonged mechanical ventilation and ventilator-associated pneumonia. Our primary objective in this study was to evaluate an ETT-clearing device (ETT-CD) in its ability to remove secretions from *ex vivo* ETT lumens.

**Methods:**

Forty ETTs, obtained from intensive care patients at extubation, were individually placed into a ventilator field performance testing simulator at 37°C. The pressure drop through the ETTs was measured at a flow rate of 60 L/min before and after cleaning with the ETT-CD and compared with unused, similarly sized controls tubes. The ETT-CD was inserted into an ETT until the tip reached Murphy's eye (hole in the side) of the ETT. The wiper, set back from the tip, was expanded by ETT-CD handle activation. As the ETT-CD was removed, the distal wiper extracted secretions from the ETT lumen.

**Results:**

Forty ETTs were tested with nonparametric Wilcoxon signed-rank tests. Before being cleared with the ETT-CD, the median pressure drop in the extubated 7.5 mm ETTs was 17.8 cm H_2_O; after ETT-CD use, it was 12.3. The cleared ETTs were significantly improved over the ETTs before being cleared (*p* < 0.001); however, there remained a significant difference between the cleared ETTs and the control tubes (*p*=0.005), indicating the clearing was not to the level of an unused ETT. Similar results were determined for the 8.0 mm ETTs.

**Conclusions:**

For the 7.5 mm and the 8.0 mm EETs, the ETT-CD improved effective patency of the ETTs over the uncleared ETTs, independent of occlusion location, tube size, or length of tube. However, there remained a significant difference between the cleared tubes and controls.

## 1. Introduction

The physical and economic burdens of ventilator-associated pneumonia (VAP) have been well documented and recognized as major consequences of mechanical ventilation (MV). VAP has significant morbidity, mortality, and cost [1]. The financial burden and demand upon resources are between approximately $40,000 and $90,000 per mechanically ventilated patient [2]. Patients' quality of life is impacted by VAP discomfort and the burden of additional hospital stay. Depending upon how VAP is defined, it occurs in approximately 10% [3] of adults aged 65 years and above. The VAP incidence has been relatively stable and high [3]. Thus, there is a critical need to develop new preventative strategies against the development of VAP in mechanically ventilated patients.

Initially, preventative strategies for VAP were focused on the positive pressure ventilator and reducing microbial contamination with heating elements in the airway tubing [4]. Antimicrobial therapy was (and is) used routinely in the management of VAP in critically ill patients. However, due to the emergence of multidrug-resistant or extremely drug-resistant pathogens [5], alternatives to antimicrobial therapies are needed. Therefore, the focus began to change to limiting biofilm development and/or reducing biofilm load and secretions in the lumens of endotracheal tubes (ETT) through the use of noninvasive management teams to monitor patients on MV with daily oral infection control [6]. The concept is valid, although healthcare personnel compliance with such protocols is problematic [7].

The secretions, including oral microflora, create a biofilm which has the potential to accumulate above the ETT cuff. These secretions then enter the ETT proper and adhere to the ETT lumen causing a constriction [6, 8]. Constriction of the lumen results in airway resistance and pressure drop in ETT during MV.

An ETT lumen during MV is a consistent surface for microbial colonization since the ends of an ETT are under constant parallel lines of force, while the central curve of the tube experiences a vortexing action [9]. As the biofilm develops and secretions adhere, the lumen of an ETT occludes and narrows. To clear secretions from the ETT lumen, closed-system suctioning is periodically employed [10]. However, it was reported that the incomplete removal of secretions during suctioning was found to be a factor in the narrowing of an ETT's lumen [11, 12].

Secretions and biofilms accumulate quickly and unpredictably [13, 14]. When they detach from the luminal ETT, they can project deep within the lungs (up to 45 cm from the end of the ETT) [15]. It should be noted that, in addition to an ETT having secretions that can enter the lungs, oral bacteria may migrate quickly from the mouth during intubation and contribute to VAP pathogenesis [16].

ETT lumenal occlusion with biofilm and secretion, which is unpredictable of MV time [17], may result in VAP or other infections in the lungs. This should be considered when weaning is difficult, even for patients ventilated for less than one day [18]. ETT obstruction may mislead clinicians on the readiness of patients for extubation and subsequently lead to unnecessary prolongation of intubation and MV [19, 20].

In the designed study, we hypothesized that using a novel federally registered medical device to clear biofilm secretions from the ETT lumens would return the ETT to luminal patency. To test this hypothesis, we designed a study aimed at measuring the impact of an ETT clearing device (ETT-CD) (EndOclear® Restore™ device, previously named EndOclear® endotracheal tube clearing device) within controlled *ex vivo* conditions. To demonstrate an *ex vivo* model, we designed a multistation ventilator field performance testing simulator for data acquisition.

## 2. Materials and Methods

This study was conducted at West Virginia University Hospital in association with the Department of Pathology and was approved by the West Virginia University Institutional Review Board (IRB H-23234). Forty ETTs were randomly obtained from adult patients at the time of extubation from a combined Medical/Surgical Intensive Care Units (MICU/SICU). All ETTs of adult patients (age >18 years old) were eligible for collection, unless the patient was diagnosed with tuberculosis and/or HIV. Patient medical histories, dates, times of intubation and extubation, length of ETT intubation (days), and clinical and ventilator information were recorded in a database.

### 2.1. Ex Vivo Measurements

The setting, testing design, and calibration method of the pressure drop test apparatus were published previously [13]. The simulation device was modified with an engineered heat-regulated tracheal feature with a temperature sensor probe to maintain the *ex vivo* ETTs at 37°C (body temperature) while conducting the research. Additionally, the new design included the positioning of the engineered heated head at a 30° anatomically correct head position following Institute for Healthcare Improvement guidelines that would be in place during patient care. The modificated design is referred to as the multistation ventilator field performance testing simulator (Supplemental Figure 1). The setup closely duplicated the *in vivo* anatomy and temperature of a human who has an ETT in place allowing for the measure of the true impact of airway resistance of the *ex vivo* ETTs.

### 2.2. Control Tubes

Standard ETTs (Hi-Lo; Mallinckrodt, Inc; St. Louis, MO) sizes at 6.5, 7.0, 7.5, and 8.0 mm internal diameter were evaluated and used as baseline controls. Each control ETT was placed and secured, by cuff inflation, into the multistation ventilator field performance testing simulator set at 37°C. A high-efficiency particulate arresting (HEPA) air breathing filter (Iso-Gard^®^ HEPA Light 28022; Hudson RCI; Temecula, CA) was attached to the proximal end of each control tube for consistency when comparing the control tube with a patient's *ex vivo* tube, as the *ex vivo* tubes needed the HEPA filter to minimize potential exposure to biohazard material.

Pressure drop was measured by opening an air flow control valve of compressed air set to a pressure of 2109 cm H_2_O (30 pounds per square inch, psi) at a controlled rate from 0 to 100 liters per minute (L/min) over 30 seconds. Data acquisition of the mass flow rate through the mass flow meter and pressure transducer (PTS-2000; Mallinckrodt, Inc; St. Louis, MO), using specialized software (Puritan Bennett Breathlab™ PTS Respiratory Products Test Software, V. 2.0 Rev. B; Mallinckrodt, Inc), was set to 72 measurements per second. The collected pressure drop data were repeated three times, averaged, logged, analyzed, and evaluated at 30, 60, and 90 L/min for reliability (Supplemental Figure 2). We focused on 60 L/min as this flow rate achieved optimal gas exchange in most critically ill, intubated adult patients [21, 22].

### 2.3. Sample Tubes

Extubated ETTs were collected and immediately placed in a biohazard bag with sterile gauze wetted with 4 ml of sterile saline and sealed to avoid dehumidification during the transportation to the laboratory. Using the same methods as for the control tubes, the *ex vivo* ETTs were connected to the multistation ventilator field performance testing simulator set at 37°C. The initial set of pressure drop data (cm H_2_O), referred to as the before clearing data (pre), was obtained using the same method as for the control tubes.

Then, each *ex vivo* ETT was cleared with the ETT clearing device (ETT-CD) (EndOclear® Restore™ device, endOclear®, Inc., Petoskey MI), a federally registered (FR Doc. 2011–21685 Filed 8-23-11; 8:45 am) medical device. The ETT-CD was designed with a flexible central tube and a smooth disc-shaped wiper at its distal end (Supplemental Figure 3). The ETT-CD was inserted into the ETT until the tip reached Murphy's eye (hole in the side) of the ETT. The disc-shaped wiper, set back from the tip, was expanded by activating the handle of the device (Supplemental Figure 4). Once deployed, the wiper is firmly engaged with the inside walls of the ETT, in its activation mode. The ETT-CD was then removed, extracting biofilm secretions with the wiper. The insertion, positioning, and clearing of the ETT (distal end first) required approximately 8 seconds. A second set of pressure drop data (cm H_2_O), referred to as the cleared data (post), were obtained using the same method as for the control and preclearing ETTs data collection procedures.

### 2.4. Statistical Analysis

Data were organized by tube size. Pressure drops were tabulated and compared with the size-matched control tubes [13]. JMP v. 9.0.0 and SPSS version 20 (SAS Institute Inc; Cary, NC) software were used to perform data analyses. Nonparametric statistical tests were used due to sample size. Related-samples Wilcoxon signed-rank tests were used to compare the ETTs before being cleared and after being cleared. One sample (vs. median control pressure drop) Wilcoxon signed-rank tests was used to compare the control tubes with the ex vivo tubes before clearing and after clearing. Significance was accepted at an alpha of <0.05.

## 3. Results

Studied *ex vivo* ETTs were from 23 male and 17 female patients with a mean patient age of 53 years (SD ± 18). The ETTs were from 15 patients diagnosed with pneumonia, 10 with a history of smoking, 12 with COPD, 22 who had fever, and 30 who received antibiotic therapy. Eight ETTs were reintubations and 31 ETTs were from patients who were on MV over 48 hours. Respiratory failure was the most prevalent reason for intubation. The collected ETTs had been in use for a mean of 2.6 days of intubation and were composed of three sizes: 7.0; 7.5; and 8.0 mm (Table 1).

### 3.1. Before Clearing the *Ex Vivo* ETTs versus Control Tube Results

There was a significant difference between the *ex vivo* ETTs' before being cleared and the control tubes indicating obstruction in the *ex vivo* ETTs as compared with the control tubes for sizes 7.5 mm and 8.0 mm (*p* < 0.001). There was no significant difference between the size 7.0 mm *ex vivo* ETTs and the control tubes (*p*=0.068).

### 3.2. Before Clearing the *Ex Vivo* ETTs versus the Cleared *Ex Vivo* ETTs Results

The overall median pressure drop for the ex vivo ETTs (*n*=40) before clearing was 17.5 cm H_2_O (minimum = 10.2 cm H_2_O; maximum = 77.8 cm H_2_O). The overall median pressure drop after clearing was 11.4 cm H_2_O (minimum = 9.5 cm H_2_O; maximum = 16.8 cm H_2_O).

There was a significant difference in the *ex vivo* ETT lumens before being cleared and after being cleared for sizes 7.5 mm and 8.0 mm tubes (*p* < 0.001), indicating a difference in pressure drop due to lumen obstruction being cleared after the use of the ETT-CD. There was no significant difference in the 7.0 mm tubes. The results are summarized in Table 2.

### 3.3. Cleared *Ex Vivo* ETTs versus Control Tubes Results

There was significant difference in the cleared *ex vivo* ETT lumens and the control tubes for the sizes 7.5 mm and 8.0 mm (*p* < 0.001), indicating a difference remained in pressure drop between the cleared tubes and the control tubes. There was no significant difference in the 7.0 mm tubes.

The majority of the cleared ET tubes were within 5–13% of the pressure drop observed in control tubes. Utilization of the ETT-CD resulted in a 22–145% decrease in pressure drop in all ET tubes at 60 L/minute. The average percent increase in pressure drop from control tubes for the remaining flow rates at sizes 7.0, 7.5, and 8.0 mm are displayed in Supplemental Figure 5.

Pressure drop data from ETTs before clearing were compared to control tubes to assess functionality of a smaller tube size at 60 L/min. 1 of 4 ETTs (7.0 mm), 11 of 17 ETTs (7.5 mm), and 14 of 19 ETTs (8.0 mm) had a pressure drop equivalent to 1 size smaller. ETT sizes 7.5 mm and 8.0 mm presented greater pressure drops compared to controls of smaller sizes. 7 of 17 ETTs (7.5 mm) and 12 of 19 ETTs (8.0 mm) had a pressure drop equivalent to 2 sizes smaller. Detailed percentage of ET tubes functioning at smaller sizes, prior to clearing, is outlined in Supplemental Table 1.

## 4. Discussion

Forty extubated ETTs were randomly obtained from mechanically ventilated adult patients from a combined Medical/Surgical Intensive Care Units (MICU/SICU). The ETTs were placed into a simulator's anatomical trachea, duplicating *in vivo* placement, thereby creating a more realistic experimental platform. The ETT-CD improved the effective patency of cleared ETTs versus ETTs before clearing for the 7.5 mm and 8.0 mm (*p* < 0.001); however, compared with control tubes, some secretion remained (*p* < 0.001). The ETT-CD was effective in this study in improving the pressure change between the 7.5 mm and 8.0 mm ETTs before being cleared and after being cleared through the entire length of the tube, experiencing no issues in the navigation of the curvature of the simulated anatomical trachea while the head was in the 30° resting position.

Significant obstruction to respiratory flow begins within hours following tracheal intubation as a result of biofilm occlusion within the ETTs' lumen. Biofilm occlusion in the ETT lumen results from the accumulation of secretions and from the colonization of fungal and bacterial organisms [23, 24]. Researchers have shown that contaminated oropharyngeal secretions and/or gastric contents pool above the inflated ETT cuff in the subglottic space, causing microaspiration, tracheobronchial colonization, and VAP incidence [25, 26]. Leakage of subglottic secretions around the cuff (between ETT and tracheal mucosa) reach the distal airways within hours following endotracheal intubation [13, 26–30]. Once in the distal airways, contaminated secretions and biofilm can move more easily within the lungs. Biofilm, itself, acts as a nidus for inoculation in the lower respiratory tract, with the potential for VAP [26, 31, 32]. VAP is associated with prolonged hospitalizations, increased length of stay in an ICU, and increased costs.

To address these problems, a number of long established and innovative modifications to respiratory care and medical treatment practices have been developed to improve artificial airways, limit airway contamination, decrease infection rate, reduce airway resistance, return ET tube patency, and improve outcomes of mechanically ventilated patients. Currently, the institutional standard of care commonly used to clear secretions from an ETT is blind tracheal suctioning through a closed system [33, 34]. This maneuver, however, has been found to be poorly effective, as the literature has shown blind suctioning to decrease lung volume leading to hypoxia, trigger cardiac arrhythmias, increase intracranial pressure, and detach aggregates of ETT biofilm into the lower respiratory tract [26, 33]. Several researchers [33], also found a significant degree of ETT luminal obstruction in intubated patients despite optimal humidification and standard ETT suctioning.

To combat the collection of contaminated secretions above the ETT cuff, subglottic suctioning was established. Subglottic suctioning is a method that requires the use of a modified ETT with subglottic suction capability. It is recommended for patients who require more than 72 hours of mechanical ventilation [34, 35]. Subglottic suctioning has been shown to cause tracheal mucosa damage, as well as, tracheal injury immediately adjacent to the subglottic suction [22, 25, 35]. Suction dysfunction has raised concerns about safety, as dysfunction has been attributed to obstruction of the subglottic suction port by suctioned tracheal mucosa [26, 35].

In recent years, several researchers and companies have focused their efforts to develop novel medical devices (e.g., Mucus Shaver, Obstruction Remover, Mucus Slurper, and Rescue Cath), different from suctioning, aimed specifically at cleaning the ETT lumen by removing secretions attached to the ETT by physically scraping or wiping the internal surface of the ET tube [12, 25, 33]. Acoustic reflectometry, high-resolution computed tomography [33], intraluminal catheter, pressure oscillation analyses [36], sound analysis [37], and resistive work of breathing are all methods used to evaluate modified ETTs and ETT devices by researchers. Although potential benefits may exist, most studies involved partial assessments of the ETT, which in turn could potentially affect the validity and efficacy of the method. In addition, large clinical trials and laboratory studies have been limited in the testing of these medical devices as they lack accurate measurements of ETT patency and have not provided concrete evidence for wide use [12, 33, 34, 38].

Our study aimed at overcoming the shortcomings of previous trials by a more rigorous design and at developing a standardized testing procedure to measure the impact of an ETT-clearing device (ETT-CD) in returning the ETT to luminal patency within controlled *ex vivo* conditions.

Mietto et al. [12] estimated that the average loss of intraluminal ETT volume from partial occlusion due to secretion accumulation was between 9 and 15%. Our results estimated that nearly 25% of ET tubes at size 7.0 mm, 65% at size 7.5 mm, and 74% at size 8.0 mm had a measurable pressure drop equivalent to one size smaller. More apparent was the decrease in pressure drops in ETT tubes sizes 7.5 mm and 8.0 mm where 41% of 7.5 mm and 63% of 8.0 mm were estimated to function at the same value as ETTs two sizes smaller. We did not find any underlying factors associated with the increase in biofilm secretions within the ETT lumen. We did, however, find a positive correlation between the volume extracted from the ETT lumen and pressure drop observed in the multistation ventilator field performance testing simulator, independent of LOI.

Unlike Glass et al. [11], who predicted the longer the time an ETT was in place, the greater the opportunity for debris to accumulate, our findings closely matched those of Wilson et al. [13], who illustrated the unpredictable nature of ET tube obstruction and its independence of LOI. Consistent with Wilson et al.'s findings [13], a patient intubated 3 hrs or 300 hrs could have either significant increases in resistance or measurements equivalent to those of size-matched control tubes. The amount of biofilm secretions within the ETT lumen was not a time-related event [13]; hence, partial occlusion due to secretion accumulation cannot be recklessly ignored.

Our results show that *ex vivo* ET tubes, before clearing, had pronounced pressure drops (and therefore a higher degree of resistance) of 32–158% from control tubes at the 7.5 mm and 8.0 mm luminal sizes. We tested the efficacy, safety, and feasibility of the ETT-CD in an *ex vivo* model, which reduced luminal biofilm secretions of ETTs to within 5–13% of the pressure drop observed in control tubes. Not only was the ETT-CD found to be easy to use but it also was effective in removing secretions from the ETT lumens in the 7.5 mm and 8.0 mm tubes.

### 4.1. Limitations

Although the sample size (*n*=40) was adequate for statistical observations regarding device impact and clinical associations, the small sample number of size 7.0 mm ETTs (*n*=4) limits our findings for that diameter ETT; nevertheless, consistent results were found for all of the ETTs studied. Moreover, the study was not powered to evaluate the efficacy of the ETT-CD to respiratory care and medical treatment practices, such as, standard suctioning. The study was designed to evaluate the ETT-CD and its ability to remove luminal biofilm secretions and thereby increase patency of the ETT. Larger studies are needed to determine whether the use of the ETT-CD has any effect on VAP incidence, mortality, duration of MV, and ICU or hospital stay. The three ETT sizes collected and tested against the ETT-CD had internal diameters of 7.0, 7.5, and 8.0 mm because these are the ETT sizes used for adults in our MICU/SICU. Additional studies are needed to validate ETT-CD functionality in smaller ETTs, as well as, comparative studies against blind tracheal suctioning through a closed system, the institutional standard of care.

## 5. Conclusion

Within hours following tracheal intubation, ETTs in MV patients begin to accumulate luminal biofilm secretions causing narrowing or even occlusion of the ETT, as previously reported. The extent of the biofilm accumulation and occlusion in any one patient is unpredictable. The ETT-CD was designed to clear the lumen of the ETT while the tube is functioning as a conduit airway from the patient's trachea to the mechanical ventilator without the use of antimicrobials. The newly designed multistation ventilator field performance testing simulator allowed for a more representative means to evaluate the impact of any clearing device in reducing airway resistance. Following the use of the ETT-CD, the 7.5 mm and 8.0 mm *ex vivo* ETTs in the study were significantly improved over the ETTs before being cleared, independent of occlusion location, tube size, or length of tube.

## Figures and Tables

**Table 1:** Characteristics of the study population.

Variable	Mean (SD)
Age ± SD	53.02 (17.89)
Height (in.)	67.59 (4.73)
Weight (kg)	86.18 (31.13)
Hospital LOS (days)	17.5 (12.42)
ICU LOS (days)	8.8 (6.90)
Vent days	3.1 (2.49)
LOI (days) for ETTs obtained	2.60 (2.32)
*History*	*n* (% of total)
Male patients	23 (57.5%)
Female patients	17 (42.5%)
Smokers	10 (25.6%)
Patients on antibiotic therapy	30 (81.08%)
Patients on ventilator >48 hours	31 (77.5%)
*Comorbidities*	
Reintubation	8 (20.0%)
Pneumonia prior to intubation	9 (22.5%)
Pneumonia following intubation	13 (32.5%)
Total pneumonia	15 (37.5%)
History of tracheostomy	3 (7.5%)
COPD	12 (30%)
Fever	22 (59.46%)
*Reason for intubation*	
Respiratory failure	21 (52.5%)
Others (surgery, respiratory arrest, drug OD)	19 (47.5%)

**Table 2:** Pressure drop characteristics of tested and control endotracheal tubes at 60 L/minute.

Type of endotracheal tube	*n*	Mean pressure drop	Standard deviation	Min. pressure drop	Max. pressure drop	*t*-test value vs. control	*p* value	*t*-test value vs cleared tube	*p* value
*All endotracheal tubes*									
All controls	3	10.8	0.27			n/a		−8.63	<0.001
Extubated, not cleared (pre)	40	21.8	13.30	10.2	77.8	5.27	<0.001	4.80	<0.001
Extubated, cleared (post)	40	11.8	0.29	9.5	16.8	−8.63	<0.001	n/a	
*7.0 mm diameter*									
Control	1	14.5	0.14			n/a		−3.69	0.035
Extubated, not cleared (pre)	4	19.2	1.88	16.4	20.4	4.94	0.016	5.20	0.014
Extubated, cleared (post)	4	15.9	0.75	15.0	16.8	−3.69	0.035	n/a	
*7.5 mm diameter*									
Control	1	11.8	0.01			n/a		−3.30	0.004
Extubated, not cleared (pre)	17	24.1	9.12	12.0	46.0	4.33	<0.001	4.13	0.001
Extubated, cleared (post)	17	12.5	0.86	11.4	14.3	−3.30	0.004	n/a	
*8.0 mm diameter*									
Control	1	9.1	0.04			n/a		−10.76	<0.001
Extubated, not cleared (pre)	19	23.0	17.42	10.2	77.8	3.55	0.002	3.23	0.004
Extubated, cleared (post)	19	10.3	0.48	9.5	11.3	−10.76	<0.001	n/a	

## Data Availability

The SPSS data used to support the findings of this study are restricted by the West Virginia University Institutional Review Board in order to protect patient privacy. Data are available from the corresponding author, Christopher Waters, for researchers who meet the criteria for access to confidential data.

## Supplementary Materials

Supplementary Materials Supplemental Table 1: functionality of uncleared ETTs in relation to ETT size. Supplemental Figure 1: schematic representation of the multistation ventilator field performance testing simulator. The simulator is organized into four regions for quality control used in evaluating pressure drop (before and after clearing) accompanied by a step-by-step schematic representing placement of extubated ETT from 37°C “heated head” to PTS-2000. Supplemental Figure 2: measured pressure drop of each extubated patient endotracheal tube; control, before the ETT was cleared (pre), and after the ETT was cleared (post) evaluated at flow rates of (A) 30 L/min, (B) 60 L/min, and (C) 90 L/min. Supplemental Figure 3: exact representation of the endOclear endotracheal tube-clearing device (ECCD). The endOclear endotracheal tube-clearing device is composed of four components: (A) depth stop, (B) red safety toggle, (C) handle and trigger to deploy wiper, and (D) wiper, (D1) wiper closer, and (D2) wiper deployed. Supplemental Figure 4: schematic representation of the endOclear endotracheal tube-clearing device. Positioning of the ECCD in the ETT: (1) bullet-shaped tip of the flexible central tube reached Murphy's eye of the ETT, (2) the disc-shaped wiper, set back from the tip, and (3) activating handle of device deploys wiper. The ECCD is pulled back out of the tube, extracting biofilm secretions clearing the ETT. Supplemental Figure 5: comparison of the average percent (%) increase in pressure drop of ETTs (before and after clearing) from control ET tubes for each sized ETT, 7.0, 7.5, and 8.0 mm at flow rates, 30, 60, and 90 L/min.
