# Supplementary material for: Ex Vivo Evaluation of Secretion-Clearing Device in Reducing Airway Resistance within Endotracheal Tubes
Source: Crit Care Res Pract. 2018 Dec 10;2018:3258396. doi: 10.1155/2018/3258396 (PMC6311789; doi:10.1155/2018/3258396)

SUPPLEMENTAL TABLE 1. Functionality of uncleared ETTs in relation to ETT size

Tube size	Percentage of ETTs in which function associates with a smaller tube size (prior to clearing)					
	1 size smaller			2 sizes smaller		
	30 slpm	60 slpm	90 slpm	30 slpm	60 slpm	90 slpm
7 mm	0	25%	25%	Not Applicable		
7.5 mm	53%	65%	65%	29%	41%	35%
8 mm	63%	74%	79%	47%	63%	63%

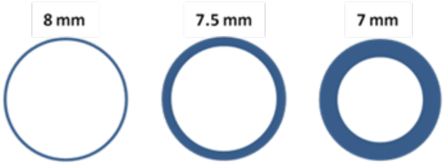

Supplemental Figure 1

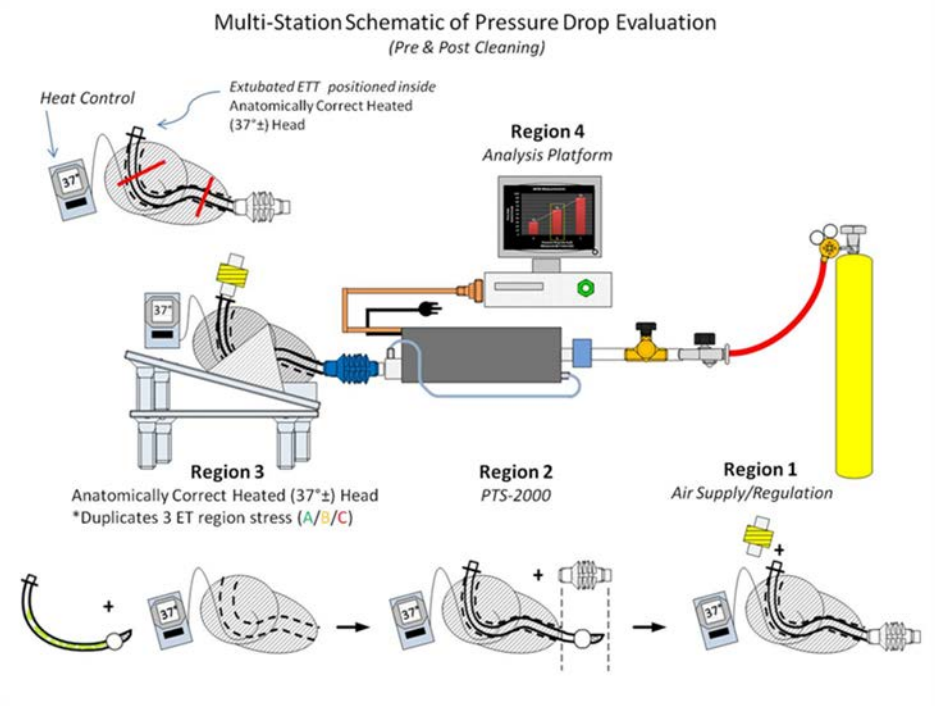

Supplemental Figure 2

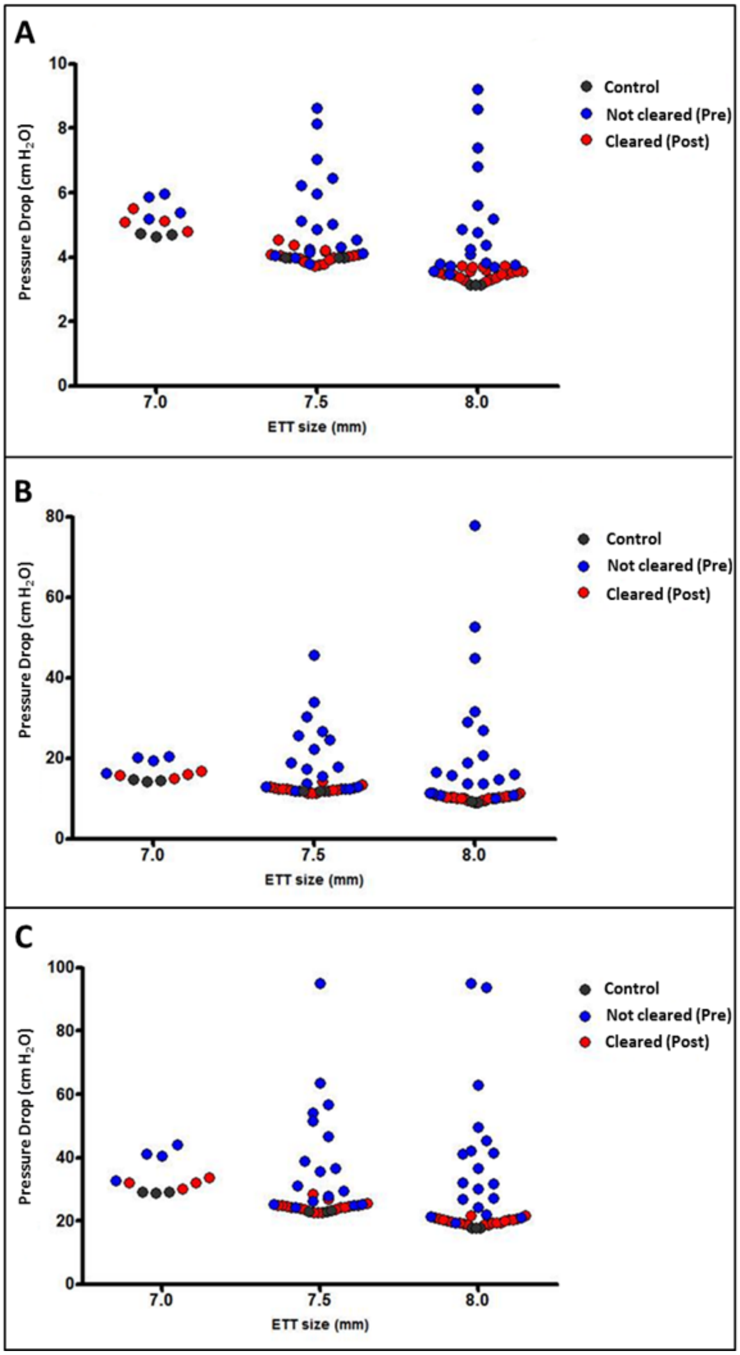

Supplemental Figure 3

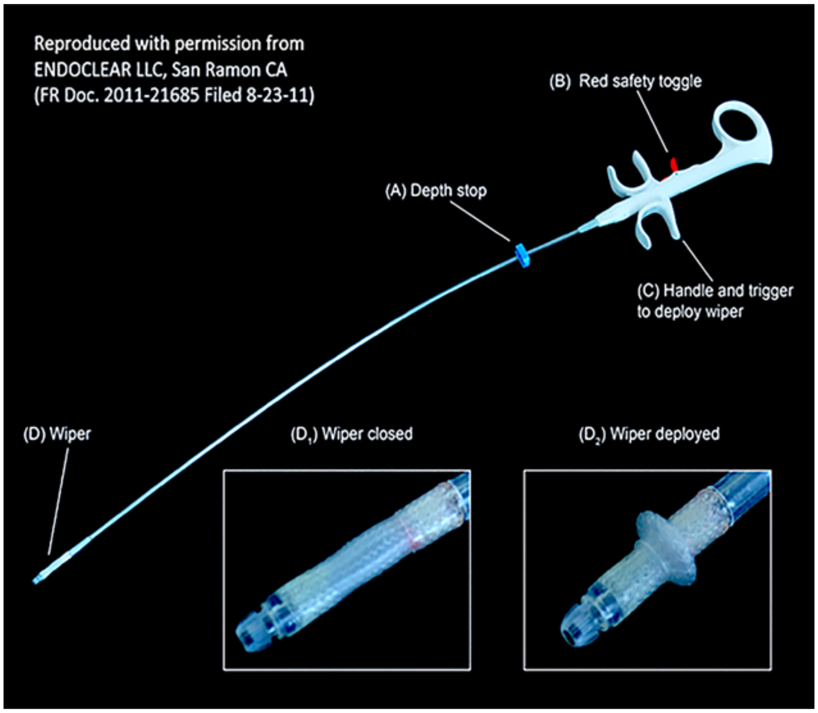

Supplemental Figure 4

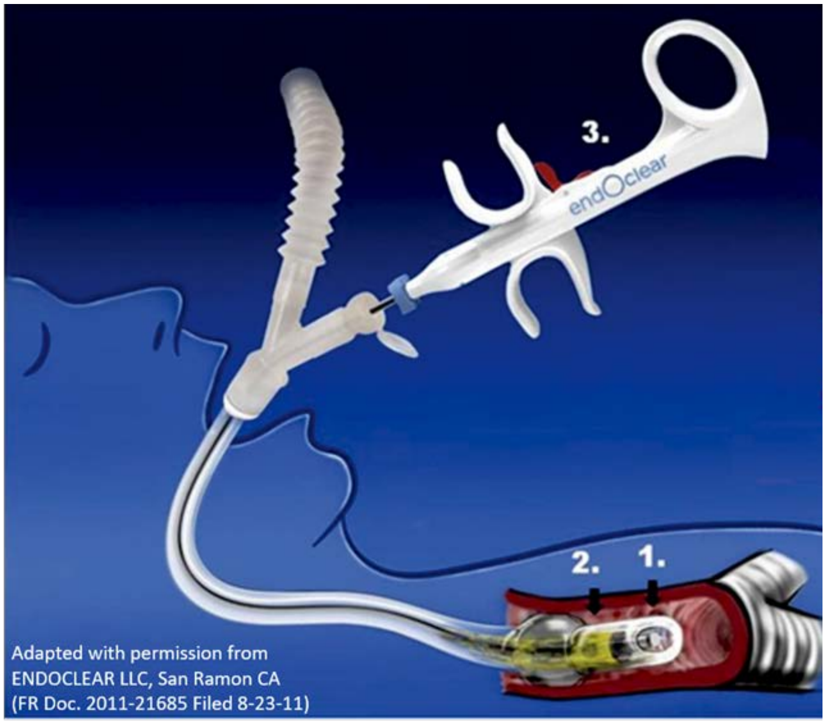

Supplemental Figure 5

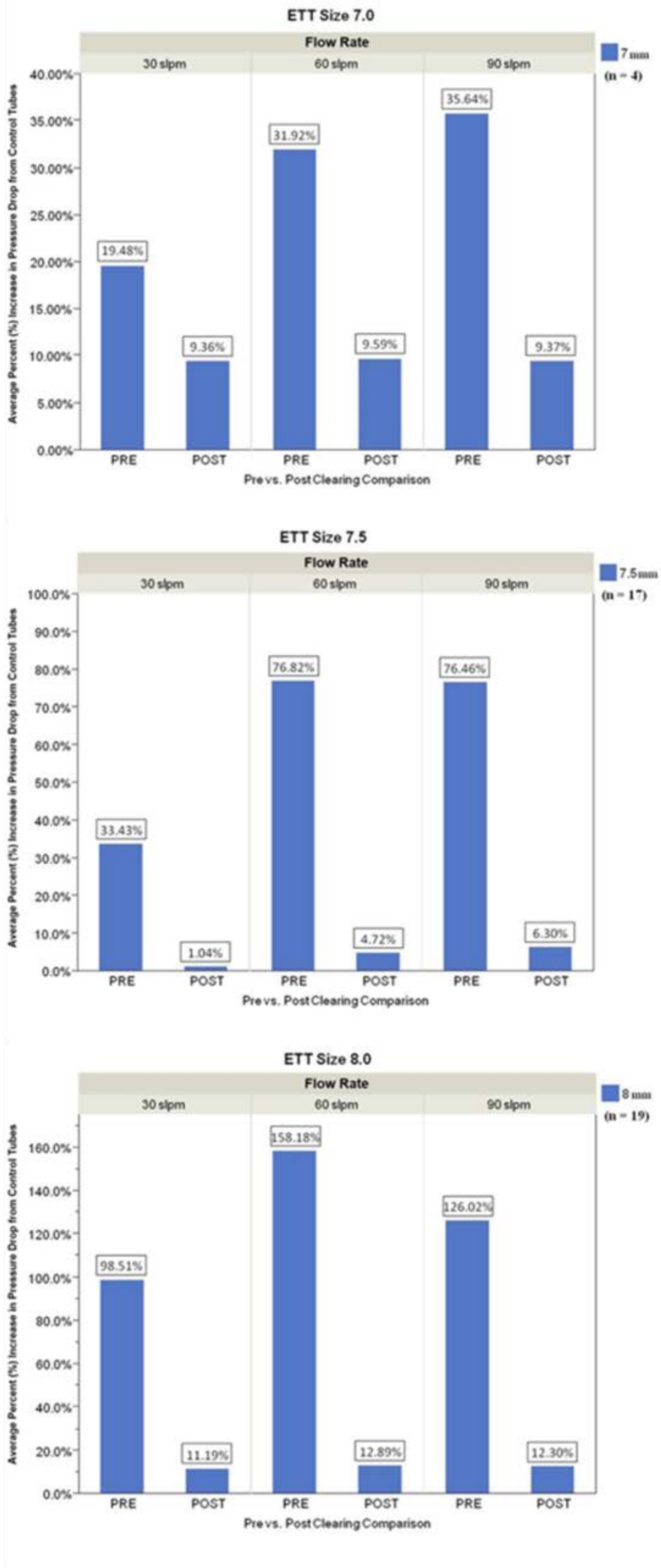

Supplement: Supplementary Materials — Supplemental Table 1: functionality of uncleared ETTs in relation to ETT size. Supplemental Figure 1: schematic representation of the multistation ventilator field performance testing simulator. The simulator is organized into four regions for quality control used in evaluating pressure drop (before and after clearing) accompanied by a step-by-step schematic representing placement of extubated ETT from 37°C “heated head” to PTS-2000. Supplemental Figure 2: measured pressure drop of each extubated patient endotracheal tube; control, before the ETT was cleared (pre), and after the ETT was cleared (post) evaluated at flow rates of (A) 30 L/min, (B) 60 L/min, and (C) 90 L/min. Supplemental Figure 3: exact representation of the endOclear endotracheal tube-clearing device (ECCD). The endOclear endotracheal tube-clearing device is composed of four components: (A) depth stop, (B) red safety toggle, (C) handle and trigger to deploy wiper, and (D) wiper, (D1) wiper closer, and (D2) wiper deployed. Supplemental Figure 4: schematic representation of the endOclear endotracheal tube-clearing device. Positioning of the ECCD in the ETT: (1) bullet-shaped tip of the flexible central tube reached Murphy's eye of the ETT, (2) the disc-shaped wiper, set back from the tip, and (3) activating handle of device deploys wiper. The ECCD is pulled back out of the tube, extracting biofilm secretions clearing the ETT. Supplemental Figure 5: comparison of the average percent (%) increase in pressure drop of ETTs (before and after clearing) from control ET tubes for each sized ETT, 7.0, 7.5, and 8.0 mm at flow rates, 30, 60, and 90 L/min. [file 3258396.f1.pdf]
